# DNA methylation landscapes in the pathogenesis of type 2 diabetes mellitus

**DOI:** 10.1186/s12986-018-0283-x

**Published:** 2018-06-28

**Authors:** Zheng Zhou, Bao Sun, Xiaoping Li, Chunsheng Zhu

**Affiliations:** 1 Department of Chinese Medicine, The First Affiliated Hospital of Zhengzhou University, Zhengzhou, 450000 China; 2 Department of Clinical Pharmacology, Xiangya Hospital, Central South University, Changsha, 410000 China; 3 Hunan Key Laboratory of Pharmacogenetics, Institute of Clinical Pharmacology, Central South University, Changsha, 410000 China

**Keywords:** T2DM, DNA methylation, Insulin secretion, Insulin resistance, Insulin-sensitive organs

## Abstract

Although genetic variations and environmental factors are vital to the development and progression of type 2 diabetes mellitus (T2DM), emerging literature suggest that epigenetics, especially DNA methylation, play a key role in the pathogenesis of T2DM by affecting insulin secretion of pancreatic β cells and the body’s resistance to insulin. Previous studies have elucidated how DNA methylation interacted with various factors in T2DM pathogenesis. This review summarized the role of related methylation genes in insulin-sensitive organs, such as pancreatic islets, skeletal muscle, liver, brain and adipose tissue, as well as peripheral blood cells, comparing the tissue similarity and specificity of methylated genes, aiming at a better understanding of the pathogenesis of T2DM and providing new ideas for the personalized treatment of this metabolism-associated disease.

## Background

Type 2 diabetes mellitus (T2DM), characterized by a complex, multifactorial, and chronic condition that often necessitates the use of various medications to achieve normal blood glucose, is a complex endocrine and metabolic disorder with dire consequences for human health and well-being. Globally, the estimated prevalence of T2DM is 415 million people in 2015 worldwide, and this figure is projected to rise to 642 million people by 2040 [1]. Significant advances have been made over the past few decades in the understanding of glucose homeostasis and the pathophysiology of T2DM [2]. However, elaborate molecular mechanisms for its pathology remains far from clear.

Increasing evidence showed that the interaction between several genetic and environmental factors contributed to the risk of developing T2DM by causing certain degrees of insulin resistance and pancreatic β-cell dysfunction [3]. Candidate approaches localized several disease genes, such as transcription factor 7 like 2 (TCF7L2) [4]. Genome-wide association studies and meta-analysis provided new insight into the genetic architecture of T2DM [5, 6]. However, although over 100 genetic loci had been identified, they collectively explained 10% susceptibility to T2DM, implying other possibilities influencing the nature of disease [7]. Epigenetics, in particular DNA methylation, was also implicated in the pathogenesis of T2DM and other complex metabolism-associated diseases by altering the expression of genes [8–10]. Even more, epigenetics built a molecular link between environmental factors and T2DM [9]. This review focused on DNA methylation landscapes in such insulin-sensitive organs as pancreatic islets, skeletal muscle, liver, kidney, brain and adipose tissue, as well as peripheral blood cells (Table 1).

**Table 1:** DNA methylation alterations in T2DM

Genes/Susceptibility factors	Methylated status	Insulin-sensitive organs	Effects	References
PPARGC1A	Hypermethylation	Human pancreatic islets	Influenced glucose-stimulated insulin secretion	[17]
KCNQ1	Hypermethylation	Human pancreatic islets	Increased the risk of T2DM	[20]
Insulin gene	Demethylation	Human and mouse islet cell	Inhibited the function of beta cell	[21]
Insulin gene	Hypermethylation	Human pancreatic islets	Increased the levels of HbA_1c_	[22]
PDX-1	Hypermethylation	Human pancreatic islets	Associated with the insulin secretion	[23]
GLP1R	Hypermethylation	Human pancreatic islets	Positively associated with BMI and HbA_1c_	[24]
MEG3-DLK1 microRNA	Hypermethylation	Human pancreatic islets	Caused the increase of β cell apoptosis	[25]
Ageing	Hypermethylation	Rat pancreatic islets	Associated with molecular inflammation	[26]
NDUFB6	Hypermethylation	Human skeletal muscle	Influenced insulin sensitivity	[35]
COX5a	Hypermethylation	Rat skeletal muscle	Associated with mitochondrial dysfunction	[36]
COX7A1	Hypermethylation	Human skeletal muscle	Associated with glucose uptake in vivo	[37]
Gastric bypass surgery	Hypomethylation	Human skeletal muscle	Remodeled the promoter methylation of PGC-1α and PDK4	[38]
Acute exercise	Hypomethylation	Human skeletal muscle	Activated contraction-induced gene	[39]
Ionizing radiation	Hypermethylation	Mice skeletal muscle cells	Increased the risk of insulin resistance	[40]
Insulin and glucose exposure	Hypermethylation and hypomethylation	Human skeletal muscle	Altered the DAPK3 methylation	[41]
Gck	Hypermethylation	Rat liver	Involved in the development of insulin resistance	[42, 43]
TNFα	Hypermethylation	Mice liver and adipose tissue	Associated with the reduction of inflammation	[44]
Metformin transporter genes	Demethylation	Human liver	Improved hyperglycaemia and obesity	[45]
In-utero malnutrition	Hypermethylation	Mice liver	Influenced the expression of lipogenic genes	[46]
Loss of MBD2	Hypermethylation	Mice liver	Protected mice from insulin resistance	[52]
IGFBP1 and IGFBP7	Hypermethylation	Human peripheral blood	Associated with insulin resistance	[53, 54]
Alu repeats	Hypermethylation	Human peripheral blood leukocytes	Associated with insulin resistance	[59]
LINE-1	Hypomethylation	Human peripheral blood	Increased the risk of metabolic worsening	[60]
MCP-1	Hypomethylation	Human peripheral blood	Increased the serum MCP-1 level	[61]
TCF7L2	Hypermethylation	Human peripheral blood	Positively associated with fasting glucose	[62]
TXNIP	Hypomethylation	Human peripheral blood	Increased HbA_1c_ and fasting glucose	[65]
NR4A1	Hypomethylation	Human peripheral blood	Decreased the blood glucose	[66]
Aging	Hypermethylation	Human brain and blood	Remodeled DNA methylation	[67]

## DNA methylation

DNA methylation refers to the regulation of gene expression in the presence of impeccable DNA sequences with its patterns set up by DNA methyltransferases (DNMT), including DNMT3A and DNMT3B in early development. More than half of the genes in vertebrate genomes are associated with cytosine-phosphate-guanine (CpG) islands, which is related to the activity of gene transcription [11]. Thus, DNA methylation has been suggested as a natural integrator of genetic susceptibility and environmental exposure in common disease by playing a key role throughout life in tissue specific gene regulation and transcription [12, 13].

**Fig. 1:**
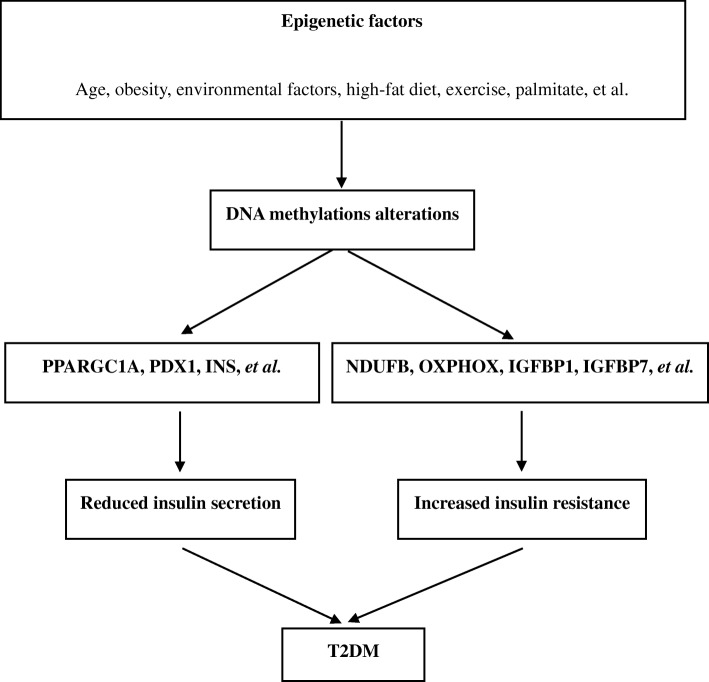
DNA methylation alterations affected insulin secretion and insulin resistance in the development of T2DM

Intriguingly, DNA methylation was found more abundant in gene bodies and often positively correlated with gene expression in recent years [14]. Besides, Aran et al. discovered that DNA methylation of enhancers could regulate transcription levels of distal promoters through binding of transcription factors and chromatin modulating proteins [15]. Additionally, genome-wide studies of the methylome had identified that the position of the methylation in the transcriptional site influenced its relationship to gene control. As a result, in the growing incidence of T2DM, DNA methylation alterations might be involved in its pathogenesis by affecting insulin secretion of pancreatic β cells and the body’s resistance to insulin across multiple tissues (Fig. 1).

## DNA methylation and T2DM

### DNA methylation alterations in pancreatic islets of T2DM

Pancreatic islets, mainly secreting insulin and glucagon, is composed of a group of hormone-secreting cells. Among them, pancreatic β cells are of utmost importance to regulate glycemia during the development of T2DM. Hence, it is essential to know mechanisms that affect the function of β cells in depth for finding new treatment options for T2DM.

Peroxisome proliferative activated receptor gamma coactivator 1 alpha (PPARGC1A), a transcriptional co-activator encoding PGC1α, was aberrantly expressed and related to impaired oxidative phosphorylation in skeletal muscle from patients with T2DM [16]. However, increased levels of methylation of the PPARGC1A gene promoter were similarly associated with its reduced expression in human pancreatic islets from T2DM patients, influencing glucose-stimulated insulin secretion [17]. The T2DM risk gene potassium voltage-gated channel subfamily Q member 1 (KCNQ1) was identified to be associated with impaired islet function [18, 19]. Travers et al. suggested that hypermethylation of the imprinted 11p15.5 region of KCNQ1 might be mediated by its genetic risk variants in both fetal pancreas and adult human islets tissues [20]. As a master regulator of metabolic homeostasis, insulin gene might be regulated by DNA methylation [21]. Yang et al. demonstrated that hypermethylation of the insulin promoter in pancreatic islets from T2DM patients was correlated negatively with insulin gene expression and positively with glycosylated hemoglobin (HbA_1c_) levels [22]. Pancreatic duodenal homeobox 1 (PDX-1) was a transcription factor that exhibited decreased expression in human pancreatic islets and was associated with insulin secretion, which was significantly associated with the hypermethylation of its promoter and enhancer regions [23]. Glucagon-like peptide 1 (GLP-1), an incretin hormone, enhanced insulin secretion by binding to its receptor (GLP1R) in pancreatic β cells. Hall et al. showed that hypermethylation of GLP1R in human pancreatic islets had positive correlation with body mass index (BMI) and HbA_1c_ of T2DM patients [24]. Moreover, another high-throughput study by Kameswaran et al. supported that MEG-DLK1 microRNA cluster was highly expressed in β cells, but repressed in human islets from T2DM donors, which was identified to be regulated by epigenetic modification [25]. Recently, Sandovici et al. suggested that age-associated transcriptional differences were negatively correlated with the methylation of several inflammation-related gene promoters in rat pancreatic islets, shedding a new light on epigenetic signatures in islets [26]. Moreover, perturbations in the intrauterine environment increasing the susceptibility of age-related diseases induced alterations in DNA methylation and gene expression in pancreatic islets of rats [27]. Meaningfully, the phenotype of T2DM was found to be potentially affected by 19 diabetes-associated single-nucleotide polymorphisms (SNPs) on which a CpG site was introduced or removed using pancreatic islets from T2DM donors, establishing the relationship between genetic variation and epigenetics [28].

With the development of high throughput sequencing, DNA methylation profiling was performed in pancreatic islets from T2DM patients. Volkmar et al. uncovered 276 CpG loci displaying differential DNA methylation by performing comprehensive DNA methylation profiling in human pancreatic islets [29]. Another genome-wide DNA methylation analysis identified 1649 CpG sites and 853 genes with differential DNA methylation in human islets. Further functional analyses found several new methylated target genes with altered expression that contributed to impaired insulin and glucagon secretion [30]. Olsson et al. demonstrated that genome-wide genetic and epigenetic variation interacted to influence gene expression in human islets [31]. Recently, whole-genome bisulfite sequencing by Volkov et al. provided a picture of DNA methylome in human pancreatic islets and stressed the importance of epigenetic changes in the pathogenesis of T2DM [32]. Interestingly, palmitate treatment of human pancreatic islets gave rise to DNA methylation modifications, which might cause impaired insulin secretion and aggravated T2DM [33]. Lately, aging increased DNA methylation of 241 sites that were identified in human islets and associated with insulin secretion [34].

### DNA methylation modifications in skeletal muscle

As an important insulin-sensitive organ, skeletal muscle was the main tissue responsible for glucose uptake. DNA methylation modifications in skeletal muscle might contribute to insulin resistance, which was beneficial for understanding the pathogenesis of T2DM.

Studies found that increased DNA methylation in the promoter of ubiquinone oxidoreductase subunit B6 (NDUFB6) was correlated negatively with its expression in skeletal muscle from T2DM patients, which subsequently influenced insulin sensitivity [35]. Gong et al. observed that hypermethylation of cytochrome c oxidase 5a (COX5a) was related with mitochondrial dysfunction in skeletal muscle of high-fat diet (HFD) induced rats [36]. Furthermore, Rönn et al. provided evidence that age was a factor influencing DNA methylation of COX7A1 that encoded oxidative phosphorylation (OXPHOS) gene in human skeletal muscle [37]. They found that the methylation of the COX7A1 promoter was increased in muscle from elderly compared with young twins. Besides, Roux-en-Y gastric bypass could remodel the promoter methylation of genes in skeletal muscle. Barres et al. revealed that the methylation of PGC-1α and pyruvate dehydrogenase kinase 4 (PDK4) was reduced with obesity and restored to nonobese levels after Roux-en-Y gastric bypass [38]. Analogously, acute exercise also could decrease promoter methylation and trigger structural and metabolic adaptations in skeletal muscle [39]. Whole genome methylation study indicated that the promoter methylation of PGC-1α, PDK4 and PPAR-δ was dramatically decreased in mouse soleus muscles [39]. Recently, Nylander et al. revealed that ionizing radiation altered substantial DNA methylation in skeletal muscle and adipose progenitor cells, increasing the risk of insulin resistance and T2DM [40]. Additionally, insulin and glucose exposure altered the DNA methylation profile and modulated death-associated protein kinase 3 (DAPK3) DNA methylation in skeletal muscle from T2DM patients [41].

### DNA methylation changes in liver and adipose tissue

Liver and adipose tissue were essential for maintaining the balance of glucose homeostasis, being the important endocrine system affecting many metabolic pathways.

Earlier study by Jiang et al. demonstrated that hypermethylation of hepatic glucokinase (Gck) contributed to diabetogenic potential in the liver from aging rats [42]. They also indicated that hypermethylation level of Gck and L-type pyruvate kinase (LPK) promoters was involved in the development of insulin resistance in high-fat diet-induced obese rats [43]. Of note, helichrysum and grapefruit extracts increased DNA methylation of TNFα in the liver and adipose tissue from diabetic mice, which might be associated with the reduction of inflammation in diabetes and obesity [44]. Metformin decreased DNA methylation of metformin transporter genes in the human liver, which might be conducive to improving hyperglycaemia and obesity [45]. Besides, in-utero malnutrition resulted in DNA methylation modifications in germ cells, which subsequently influenced the expression of lipogenic genes in livers of offspring mice [46]. Of late years, genome-wide methylome and transcriptome analysis highlighted that epigenetic and transcriptional changes in the liver from patients with T2DM played an important role in its etiology [47, 48]. In addition to that, subjects with T2DM also exhibited transcriptional and epigenetic changes in adipose tissue relevant to the development of the disease [49]. By using the Infinium HumanMethylation450 BeadChip assay, Rönn et al. stressed that increased age, BMI and HbA_1c_ levels had a great influence on epigenetic variation of candidate genes of T2DM in human adipose tissue [50]. Another genome-wide DNA methylation research revealed that patients were predisposed to insulin resistance and T2DM with aberrant DNA methylation in the visceral adipose tissue [51]. Recently, study by Cheng et al. discovered that loss of methyl-CpG-binding domain 2 (MBD2) resulted in DNA methylation changes associated with altered energy homeostasis, which protected mice from HFD-induced obesity and insulin resistance [52].

### DNA methylation shifts in peripheral blood cells

Although DNA methylation in tissues had been widely studied, it was difficult for some human tissues such as pancreatic islets, liver, skeletal muscle and adipose tissue to be non-invasively assessed. Thus, blood might be considered as a fitting surrogate marker for reflecting DNA methylation in tissues. Gu et al. demonstrated that the methylation levels of insulin-like growth factor-binding protein 1 (IGFBP1) and IGFBP7 were increased in peripheral blood from Swedish men with newly diagnosed T2DM [53, 54]. Genome-wide survey uncovered that low methylation level in human peripheral blood was an early marker of T2DM [55]. Specifically, global DNA methylation analysis unraveled that methylation level was increased in B cells and natural killer cells, but unchanged in monocytes, lymphocytes or T cells from obese and T2DM patients [56]. del Rosario et al. also supported that DNA methylation shifts in peripheral blood leukocytes of mothers who had diabetes during pregnancy were associated with increased risk for T2DM of their offspring [57]. Beyond that, premature DNA methylation aging in peripheral blood leukocytes was also correlated with increased risk of T2DM [58]. A monozygotic twin study showed that insulin resistance was probably associated with global DNA methylation of Alu repeats in peripheral blood leukocytes [59]. In addition, a cohort study suggested that hypomethylation of long interspersed nucleotide element 1 (LINE-1) in peripheral blood was correlated with a higher risk metabolic status worsening [60]. Like some T2DM-associated susceptibility gene methylated in tissues, several risk genes also exhibited methylation changes in peripheral blood from T2DM patients [61, 62]. Hypomethylation of monocyte chemoattractant protein-1 (MCP-1) promoter region in the peripheral blood mononuclear cells (PBMC) was correlated to the serum MCP-1 level, HbA_1c_ and fasting blood glucose in T2DM patients [61]. Moreover, abnormal methylation of TCF7L2 promoter was further related to fasting glucose in peripheral blood from T2DM patients [62]. Epigenome-wide association study found five methylation markers that were associated with future T2DM incidence, including ATP binding cassette subfamily G member 1 (ABCG1), phosphocholine phosphatase (PHOSPHO1), suppressor of cytokine signaling 3 (SOCS3), sterol regulatory element binding transcription factor 1 (SREBF1) and thioredoxin interacting protein (TXNIP), throwing new lights on the pathogenesis underlying T2DM [63]. Subsequently, Dayeh et al. validated the five genes in blood DNA of subjects from the prospective study, but only found the association of ABCG1 and PHOSPHO1 methylation with the risk of T2DM [64]. Furthermore, Florath et al. investigated that decreased methylation levels at cg19693031 located in the 3′-untranslated regions of TXNIP might increase fasting glucose and HbA_1c_ concentrations in T2DM patients [65]. Recently, Chen et al. showed that reversing the methylation of the nuclear receptor subfamily 4 group A member 1 (NR4A1) contributed to its high expression and decreased blood glucose in T2DM patients [66].

In addition to the above insulin-sensitive organs or tissues, recent study reported aging effects on DNA methylation modules in human brain and blood tissue, which signified that DNA methylation shifts might also exist in brain or other specific tissues from T2DM patients [67]. Also, the desensitization of insulin receptors in the brain might be regulated by epigenetic mechanisms that played a pivotal role in the pathogenesis of both Alzheimer’s disease and T2DM [68].

## Conclusion and future perspective

Novel research in the field of epigenetics opened up new sights into the pathogenesis of T2DM and provided further opportunities of identifying biomarkers for this complex metabolic disease. Epigenome-wide as well as site specific DNA methylation association studies were rapidly revealing different methylated genes in insulin-sensitive organs or tissues from T2DM patients. Even more, some certain methylation sites were introduced or removed by the mutation of T2DM susceptibility genes, partially elucidating the molecular mechanisms through which the T2DM SNPs affected gene function via differential DNA methylation [28]. In the last year, mechanistic studies of DNA methylation regulation indicated the correlation between cellular signaling pathways and DNA methylation [69], thereby increasing the possibilities to understand the etiology of T2DM and leading to a personal treatment for this disordered disease.

Current data supported the view that DNA methylation changes in different relevant metabolic tissues such as pancreatic islets, skeletal muscle, liver, brain, adipose tissue and PBMCs might be involved in the pathogenesis of T2DM. It was interesting to find that DNA methylation modifications in several certain genes were common within human tissues [70, 71]. Hence, DNA methylation in blood, a potential surrogate for other hard-to-get tissues, might be promising biomarkers for T2DM. Lifestyle changes might modify DNA methylation and thus gene expression during the development of T2DM. Targeted researches brought us closer to a better understanding of the epigenetic signatures that were associated with either age- or lifestyle-related phenotypes, which might be helpful for identifying new therapeutic targets for improving insulin sensitivity [67, 72, 73].

As a common complex metabolic disorder, the detailed mechanisms of T2DM remained obscure. Day et al. proposed that a simple epigenetic drift model was insufficient to explain age-dependent methylation in tissues from T2DM patients [70]. Genetic and epigenetic study, recently reviewed by Kwak and his colleague, discussed how genetic, environmental factors and epigenetics interacted in the pathogenesis of T2DM [74]. Even more, an integrated T2DM knowledgebase that could access aggregated large-scale genetic data was appealed for advancing complex disease research [75]. Given this, large-scale epigenetic information was also required to form a database to facilitate our understanding of T2DM biology and improve patient treatment and outcomes.

In conclusion, DNA methylation provides an efficient tool for the recognition and interpretation of T2DM. Taking the role of DNA methylation in the pathogenesis of T2DM into account may contribute to a more personalized application of therapy for metabolic diseases in the future.

## Abbreviations

ABCG1: ATP binding cassette subfamily G member 1
BMI: Body mass index
COX5a: Cytochrome c oxidase 5a
CpG: Cytosine-phosphate-guanine
DAPK3: Death-associated protein kinase 3
DNMT: DNA methyltransferases
Gck: Glucokinase
GLP-1: Glucagon-like peptide 1
HbA_1c_: Glycosylated hemoglobin
HFD: High-fat diet
IGFBP1: Insulin-like growth factor-binding protein 1
KCNQ1: Potassium voltage-gated channel subfamily Q member 1
LINE-1: Long interspersed nucleotide element 1
LPK: L-type pyruvate kinase
MBD2: Methyl-CpG-binding domain 2
MCP-1: Monocyte chemoattractant protein-1
NDUFB6: Ubiquinone oxidoreductase subunit B6
NR4A1: Nuclear receptor subfamily 4 group A member 1
OXPHOS: Oxidative phosphorylation
PBMC: Peripheral blood mononuclear cells
PDK4: Pyruvate dehydrogenase kinase 4
PDX-1: Pancreatic duodenal homeobox 1
PHOSPHO1: Phosphocholine phosphatase
PPARGC1A: Peroxisome proliferative activated receptor gamma coactivator 1 alpha
SNPs: Single-nucleotide polymorphisms
SOCS3: Suppressor of cytokine signaling 3
SREBF1: Sterol regulatory element binding transcription factor 1
T2DM: Type 2 diabetes mellitus
TCF7L2: Transcription factor 7 like 2
TXNIP: Thioredoxin interacting protein
